# “A little (PPI) MAGIC can take you a long way” : involving children and young people in research from inception of a novel medical device to multi-centre clinical trial *Roald Dahl, James and the Giant Peach (1961)*

**DOI:** 10.1186/s40900-020-00243-0

**Published:** 2021-01-06

**Authors:** Nichola Abrehart, Kate Frost, the Young Persons Advisory Group, Roy Harris, Andrew Wragg, Derek Stewart, Hayfa Sharif, Rachel Matthews, Luca Marciani

**Affiliations:** 1 Nottingham Digestive Diseases Centre and National Institute for Health Research (NIHR) Nottingham Biomedical Research Centre, Nottingham University Hospitals NHS Trust and University of Nottingham, Nottingham, NG7 2UH UK; 2 Nottingham University Hospitals NHS Trust, Nottingham, NG7 2UH UK; 3 National Voices, 1st floor, Bride House, 18-20 Bride Lane, London, EC4Y 8EE UK

**Keywords:** Patient and public involvement (PPI), Involvement, Young persons advisory group (YPAG), Children and young people (CYP), Children and young people in research, GenerationR

## Abstract

**Background:**

There is often a great urgency to be inclusive when conducting research and to focus efforts with groups and communities that can be referred to as marginalised. This is especially the case in research concerning medical devices aimed at children and young people (CYP). Although involvement methodology has developed over the last two decades, it can be challenging to involve and engage CYP with confidence and clarity of purpose.

**Main body:**

Our aim was to provide a reflective narrative account of the involvement of CYP, over a period of 5 years, in a research project from conception of a new paediatric medical device through to practical application. We explored a model of patient and public involvement (PPI) through the Nottingham Young Persons Advisory Group (YPAG), part of the National Institute for Health Research (NIHR) GenerationR Alliance, in a NIHR funded research project.

The YPAG designed and created a model of the human gut, co-designed the Transicap™ mini-capsules and their packaging, co-produced patient information sheets, came up with the idea to disseminate through a project website and co-wrote and created animation videos. The YPAG involvement continued through the writing and award of the follow-on research grant (MAGIC2). During this process the YPAG modified the clinical study protocol insisting that all participants in the control arm were given the imaging test results as well, save for a delayed reading compared to the intervention arm.

**Conclusion:**

Involvement of the YPAG over the last 5 years, led to the development of a mutually beneficial partnership, enabling genuine knowledge exchange between researchers and CYP. This influenced the design, plans and actions of the MAGIC study and well into the subsequent MAGIC2 follow-on project. Moreover, these involvement models applied within a feasibility study setting, have enhanced the realism and pragmatism of the study, contributing to the project’s overall success.

## Plain English summary

Sharing experiences and the influence of involvement in research are important in helping make research studies better. This manuscript aimed to share our experiences of involving a group of Children and Young People (CYP) in our research. Over the past 5 years a small group of CYP across Nottingham, namely the Young Persons Advisory Group (YPAG), has worked with the ‘MAGIC’ study. This is a research project sponsored by the National Institute for Health Research aiming to develop a new medical imaging test for children suffering from long term constipation. For the researchers this was their first experience of involving children and young people in research. With time their involvement developed into a mutually beneficial partnership, enhancing the success of the research project and the experiences of those involved. From the initial idea of a new medical imaging device through to study completion, the YPAG meetings involved practical, creative, hands-on activities such as creating animations and working models of the human gut. We believe that involving CYP in research benefitted both the research study with real patient experiences as well as giving the CYP the opportunity to express those experiences and put them to good use.

## Background

Since September 2014 to date, a group of children and young people (CYP) from the National Institute for Health Research (NIHR) Nottingham GenerationR (https://generationr.org.uk/) Young Persons Advisory Group (YPAG) have been involved with the ‘MAGIC’ study. MAGIC is a research project aiming to develop a new mini-capsules medical imaging device to measure gut transit in paediatric constipation. The project is funded by the NIHR with GenerationR Alliance supporting the design and delivery of paediatric health research in the UK. The MAGIC study findings have been recently published in a separate manuscript which details the design of the new mini-capsules device and the scientific details of the feasibility clinical study [1]. The main features of the MAGIC study are summarised in Table 1.

**Table 1:** The MAGIC Study and The TransiCap™ Medical Device

The clinical background of the MAGIC study is paediatric constipation and the project was funded by a NIHR Invention for Innovation (i4i) Product Development Award grant. Constipation in children is a common and often distressing problem and is termed functional constipation when no underlying cause can be detected. Functional constipation prevalence worldwide is estimated between 4 and 36%, with 34% of children in Britain aged 4–11 years reported to have had constipation. The cost to the healthcare system is high, at ~$3.9 billion/year [2–8].	
Management of these children is difficult and based mostly on symptom reports [9, 10]. The existing method to measure gut transit time (the time it takes for food to move through the gut) involves ingesting plastic pellets and taking X-ray images [11]. However, X-ray images are unable to reveal the colon anatomy well and provide a harmful radiation dose. The research team sought to find a modern alternative to the old X-ray method by designing a similar method of measuring gut transit time but using magnetic resonance imaging (MRI). MRI has better image quality than X-rays and uses no harmful radiation and is therefore the preferred choice when imaging children.	
In this project the research team designed and manufactured new, small plastic capsules filled with liquid that can be seen by MRI. The capsules are only a few millimetres long and are easy for young people to swallow. They do not dissolve in the body but travel along the gut, where they can be imaged using a quick MRI scan. The TransiCap™ mini-capsules (now patented and trademarked) are a medical device manufactured by JEB Technologies Ltd. (UK).	
The team obtained all necessary Ethics and regulatory approvals and ran a ‘first-in-child’ feasibility clinical study of the mini-capsules in young patients with constipation and healthy controls. The study recruited 35 children between 7 and 18 years old. On day 1, 2 and 3 of the study the participants swallowed 24 mini-capsules and on day 4, 7 and 28 they had a quick 15 min MRI scan to locate the position of the mini-capsules. We confirmed that the mini-capsules can be swallowed easily by 35 young people and successfully imaged in the gut using MRI to determine their gut transit time.	
The research team were then successful in obtaining a second NIHR i4i grant award for the follow-on work, MAGIC2. MAGIC2 is a large multi-centre clinical trial across 8 hospitals in the UK recruiting 436 young constipated patients to assess if using the mini-capsules MRI gut transit time test can improve treatment success. Participants will be asked to swallow 24 mini-capsules each day for 3 days and then have an MRI scan on days 4 and 7. All participants will be randomly assigned to two groups. An intervention group will receive their gut transit time results immediately after the day 7 MRI scan; the results will thus be available to guide treatment selection. A control group will also have the day 7 MRI scan but the results will not be shared with their care team until the end of the trial; their treatment selection will not be guided by the MRI results. The results will only be delayed so that a proper comparison with the intervention group can be made. Computer software that can detect the mini-capsules semi-automatically will also be designed.	

The purpose of this Commentary is to share our case study of an unusually long involvement of a group of CYP in a medical device development project, from an early stage through to completion and to describe how this can have a positive effect on the study, the researchers involved and the YPAG.

The Nottingham YPAG group consisted of CYP aged between 8 and 18 years. The aims of the group were to advise researchers on whether their intended project and methods were acceptable for the inclusion of children, to interact with the technology designers, as well as developing relevant and appropriate language for research tools [12]. However, over time the Nottingham YPAG involvement grew far beyond this, expanding their remit as their relationship strengthened through shared endeavour. The Nottingham YPAG supported the MAGIC study as well as research across several other fields of paediatric research. For the MAGIC study researchers this was their first experience of working with a YPAG. From the onset of the MAGIC study, the researchers made a commitment to attend YPAG meetings on regular occasions, to ensure the group were involved and kept up to date, from start to finish of the project. This resulted in learning as much for the researchers as for the YPAG themselves.

### Definition and theoretical underpinnings

Treseder defined the participation of children in research as “a process by which CYP influence decisions which bring about change in themselves, their peers, the services they use and communities” [13].

The definition of involvement and participation however, are often confused and used inappropriately. In participation the partaker has no say in decision making, whereas in involvement the partaker is fully immersed in discussion, collaboration, decision making and holds parity. Brady and Graham highlighted the benefits of CYP involvement, by saying it gives them the opportunity to voice their opinions forming a collation of knowledge from the younger generation, making a contribution to their community, develop transferable skills, social skills as well as confidence, self-esteem and knowledge [12].

Numerous models described the various levels of involvement with children. Shier discussed 5 levels of involvement and decision making, including: Children being listened to; Supporting children in their views; Utilising the views of children; Children involved in decision making, and Children having equal power [14]. Likewise, Hart’s “Ladder of participation” showed the distinction between non-participation and the various degrees of participation by children, moving from manipulation, decoration, tokenism, through to informed, consulted involvement and on to shared decision making and child led initiatives [15]. Research funders commonly request involvement from the public and wider community as they recognise it strengthens the research and better reflects the reality of patients living with conditions [16–18]. In the past paediatric research might have enlisted adults as members who would communicate their interpretation of CYP’s thoughts, feelings and ideas by acting as proxies. The United Nations Convention on the Rights of the Child stressed strongly that children have a voice too, especially in matters which affect them, and therefore proxies are not needed; the children can represent themselves. The increased expectation to involve the public in research is supported by Fleming and Boeck, who noted research should directly reflect the personal priorities, concerns and therefore the actual needs of CYP [19]. Riggare et al. described PPI participants as patient advocates having experiential knowledge of their condition and should thus be treated with the same regard as other experts in their field [20, 21].

Patient and Public Involvement (PPI) can result in better design, dissemination, awareness of the study leading to changes in the care patients receive and triggering policy change [12, 18]. PPI can not only help to shape the practical details of research but also change the attitude of the researchers and medical experts themselves. However, the practice of involving CYP as collaborators in research although widely advocated, is still evolving and largely driven by academic professionals [22].

### Aims of involvement

**Fig. 1:**
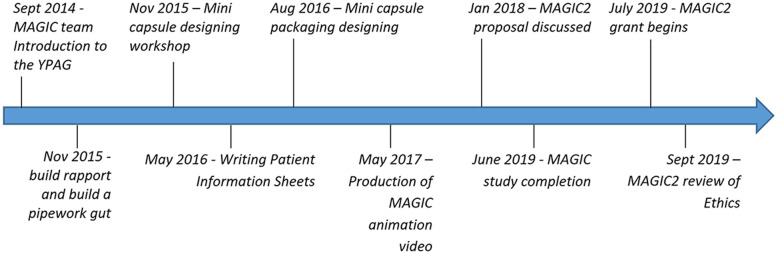
Timeline of YPAG MAGIC activities

Our initial objective was to meaningfully involve CYP to improve the design and acceptability of the MAGIC feasibility study (Table 1). We aimed to co-design the Transicap™ mini-capsules and packaging with the YPAG to ensure acceptability by our paediatric patient population. We also wanted feedback on perceptions of study outcomes, so that they reflected the priorities of CYP, as the study design included unavoidable medical procedures such as Magnetic Resonance Imaging (MRI) and the swallowing of mini-capsules on specific days. We asked the YPAG to be involved in developing information leaflets and study documentation that would be relevant to young patients and essential for study recruitment. Figure 1 below shows the timeline of activities and further details can be found in Appendix 1.

The aim of this manuscript was to share our experiences and to encourage research investigators embarking on a research project concerning CYP not just to involve them as needed but to build a long term mutually beneficial relationship.

## Main body

**Fig. 2:**
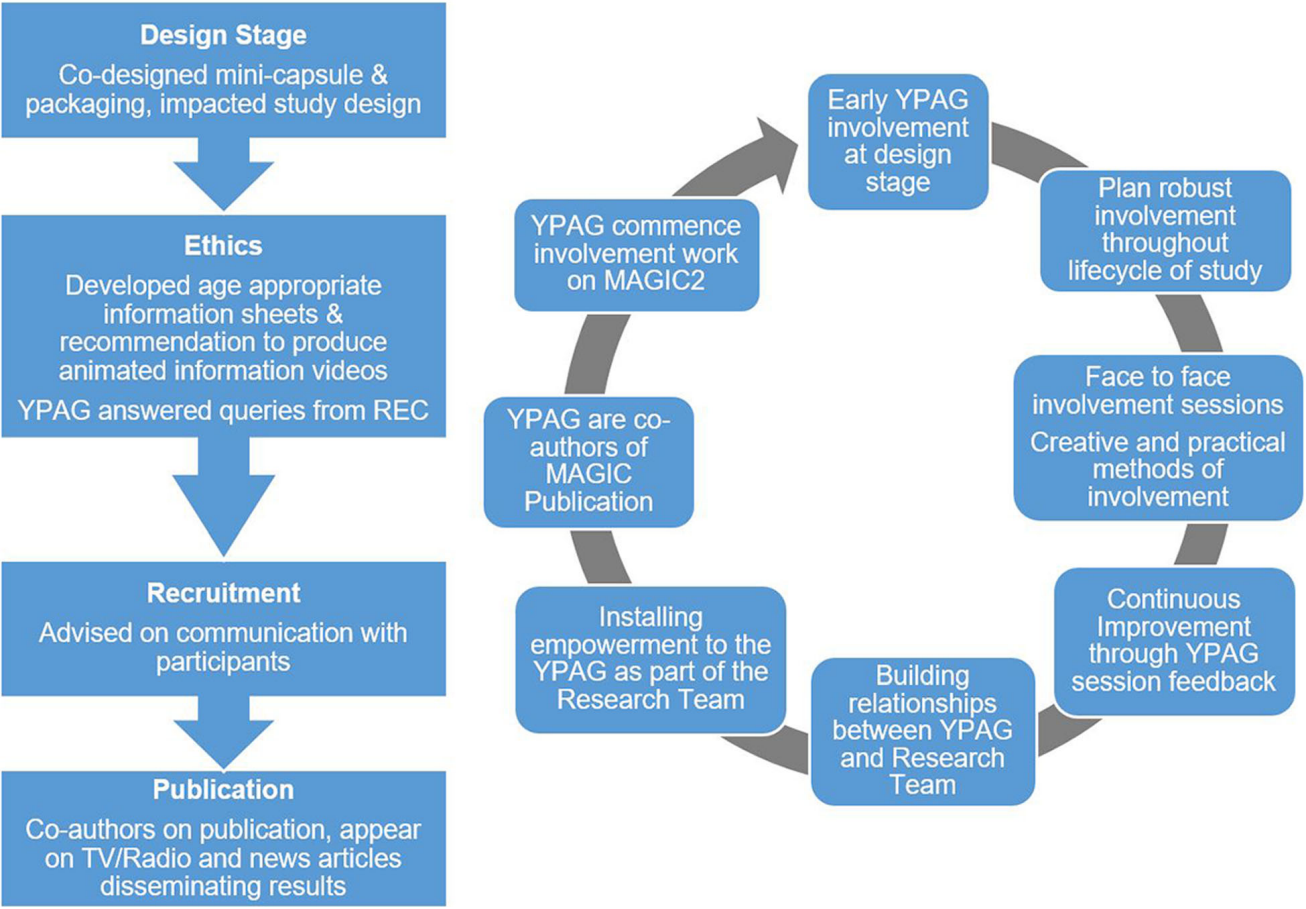
Diagram of the YPAG involvement process in the MAGIC project

Using GRIPP2 [23] as our reference guide we wished to share an account of the YPAG engagement sessions and activities the CYP took part in. The YPAG involvement process in the MAGIC project is summarised in the diagram at Fig. 2 and detailed in the subsequent paragraphs.

### YPAG meetings and members

Over the last 5 years there were 13 sessions in which the YPAG was involved in aspects of the MAGIC study and these were attended by an average of 16 CYP. Our YPAG members had a broad age range; 18% 8–11 year olds, 37% 12–15 year olds and 45% 16–18 year olds. The YPAG group consisted of a group of CYP for whom no inclusion and no exclusion criteria were set, no assumptions were made and we did not exclude anybody who wished to participate. Over the 5 years the group had a 69% retention rate. This retention rate shows the number of CYP who attended the YPAG meetings and stayed for the entire 5 years. It reflects a level of commitment of the majority of CYP, and this can be interpreted as commitment from the YPAG to paediatric research as a whole, and also a desire for personal development, which can be seen in the semi-structured interview transcript in Appendix 2. The broad age range matched the age range of the patient population which we intended to recruit into the MAGIC study. Ocloo and Matthews raise concerns that PPI groups may be insufficiently inclusive and so groups for example from black, Asian, minority and ethnic populations are kept at a distance from decision making even though those decisions may disproportionately affect them [24]. Poland et al. concurred the size of the PPI group is insignificant, the diversity of the attenders is the crucial factor to capture the varied experiences giving the researcher an insight they don’t have [25]. The members of the Nottingham YPAG were recruited widely and with no selection criteria, assumptions or interviews. Looking back at our historical membership we have indeed included members across all age range, different ethnicity and socioeconomic background, some from the general population and schools, some from out-patient clinics and some from participants to research studies. All members of the Nottingham YPAG were allowed to be involved and contribute to all the research studies put in front of them. The group consisted of CYP who were interested and wanted to make a positive contribution to paediatric research, a genuine mix of CYP brought together with a common interest in research, not a group specifically focussed on CYP with constipation.

### Safeguarding

The YPAG group leaders were adult PPI experts from Nottingham University Hospitals (NUH). All were Disclosure and Barring Service (DBS) checked, fully informed of the research projects and tasks for each YPAG session and interested in the views of young people and their involvement. All decisions concerning the functionality of the YPAG sessions were mutually agreed with the CYP. The group leaders are hereafter referred to as facilitators rather than leaders as their objective during the sessions was to guide the YPAG group rather than direct and persuade them.

### Laying the foundations for MAGIC and YPAG co-production

All activities during the YPAG sessions were specifically designed to engage the participants, encourage inclusivity and freedom of ideas. Ground rules were co-produced with the YPAG, encouraging a respectful and safe environment. Furthermore feedback after each session led to continuous development of session activities, including changes to the location of the YPAG session, from hospital based to locations within the community and University of Nottingham campus. Above all the YPAG vocalised that communication between researchers, facilitators and themselves was paramount. This is reflected in the transcript of an interview carried out as part of the YPAG’s feedback and dissemination activities in Appendix 2. The facilitator’s job during the YPAG sessions was purely to guide and support the members of the YPAG. They encouraged critical thinking, free expression of ideas and assisted discussion. The activities were engaging, child friendly, imaginative and fun. The sessions were devised by the group facilitators, but directed by the children’s own ideas of what they wanted to do. Hart commented that the limitation to research involving CYP is their competence, maturity and understanding which develops with age [26]. Our activities were adapted to ensure that every member of the group could participate regardless of their age or level of literacy or numeracy. All meetings were held at the weekend to not disrupt school life and were not scheduled too often to ensure school work, school holidays and their social life was not too affected. All children under the age of 16 were signed in and out by their parent/guardian on the day of the YPAG sessions. The consent process was developed by NUH to provide a standard and relatively simple process, whilst also ensuring parents and CYP were informed about the functionality of the YPAG. Information sheets were provided to both parents/carers and the CYP, who both gave consent and also consented for use of photos and media materials for dissemination. Full support was available if any queries during the process and the participation. Further, each YPAG participant provided evidence of allergies in relation to the catering we provided at each meeting, plus information relating to any medications they needed to take and emergency contact telephone numbers.

### YPAG tasks – the ‘pipework gut’

The first task the YPAG group approached was to consider the structure of the gastrointestinal tract and the journey the mini-capsules would be taking after the participants of the study had swallowed them. To ‘tune in’ we asked the group to build a marble run as a full scale ‘pipework gut’ using a variety of drain pipes, connectors and supports from a hardware store. This enabled the group to learn how food moved from the start (mouth) to the end (bottom) and what might prevent normal flow. The rationale behind this activity was to introduce the MAGIC study and what happens during constipation and to ensure everyone from any age within the group could fully understand the physiology of the human gastrointestinal tract. We also wanted to produce a 3D model which could be used for future demonstrations. This task also acted as an ice breaker for the YPAG and the MAGIC study research team. This was a hands-on task, where cooperation was key to the construction of the ‘pipework gut’, whereby researchers and YPAG group members worked side by side to build the final structure. Non Latin/Greek anatomical language was used during this task to promote inclusivity of all members of the team. The ‘pipework gut’ has been subsequently used at several public engagement events, including the ‘Wonder’ event of May 2019 for the University of Nottingham. As a research team we now try to find more imaginative ways of helping people understand research.

### YPAG tasks – design of the mini-capsules device

Their second task in hand was to help with the design of the mini-capsules. The YPAG group were asked to consider the size, shape of the capsules and the method in which they would prefer to swallow them.

The designing of the mini- capsules and the packaging was done in collaboration with medical device designers Renfrew Group International and the research team. The feedback from the device designs consultation with the YPAG lead us to develop a smaller, smoother, more rounded mini-capsule design. The YPAG agreed special tablet shapes, in the shape of characters, hearts, flower shapes etc., which might be more appealing to children, could prove difficult to swallow and would not be preferred and also the capsules must remain flavourless. The YPAG gave further instruction from their own personal experiences, by commenting on the method children should swallow the capsules, by using fruit juices or milk to mask the unfamiliar taste and texture of the capsules.

### YPAG tasks – design of the mini-capsules packaging

**Fig. 3:**
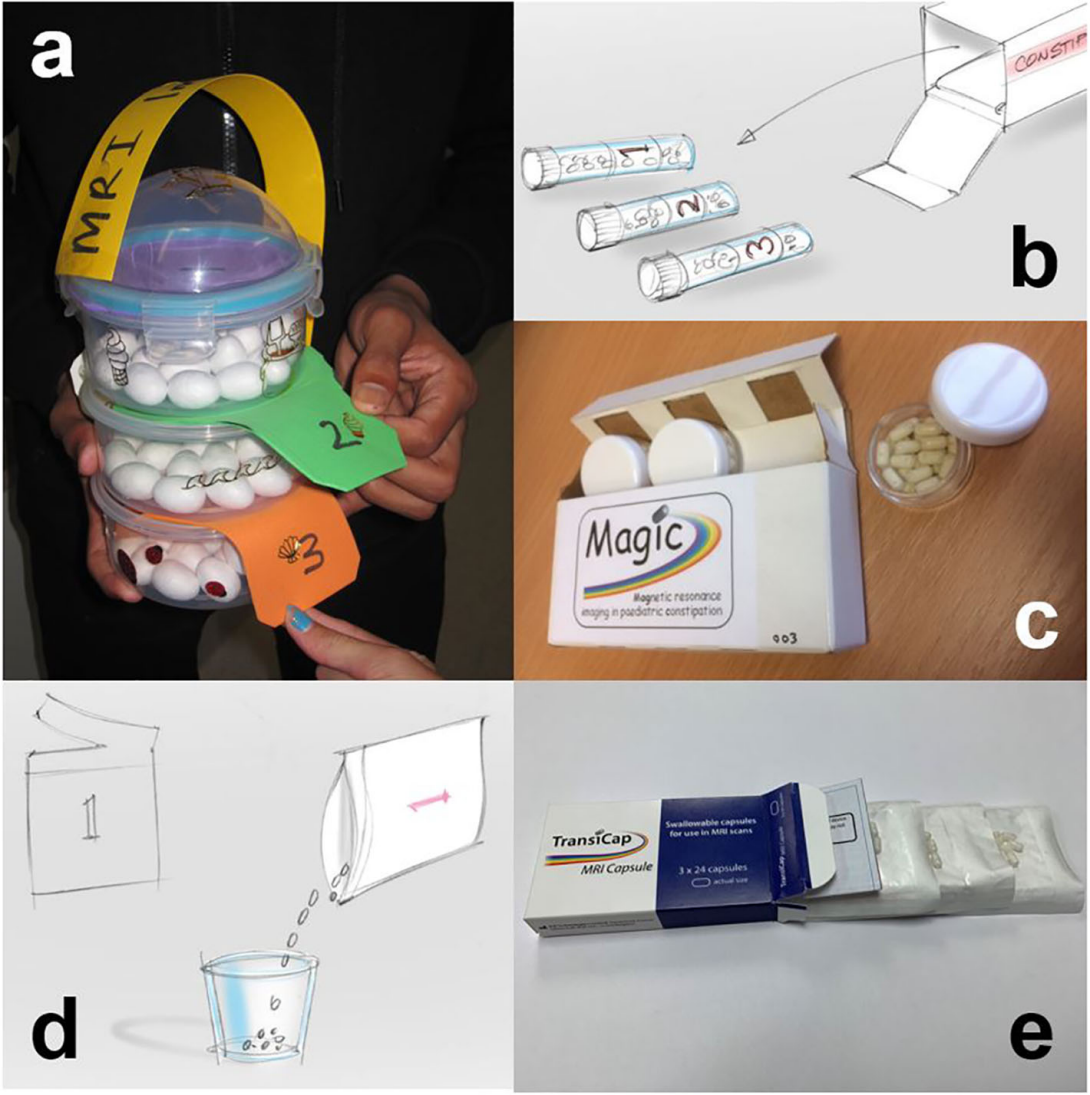
Examples of translation of YPAG ideas of mini-capsules’ packaging through designers’ sketches and to real manufacturing prototypes. **a** YPAG’s play-for-learning suggestion to use three separate round pots, **b** the designers’ sketch of what this could look line with cardboard box and **c** the corresponding real packaging prototype developed by the manufacturer. This type of packaging was further developed and went into actual use in the feasibility study. **d** An alternative YPAG suggestion was to use sachets represented with the designers’ sketch in (**d**). This design evolved into the real packaging (**e**) which is to be used in the follow-on clinical trial MAGIC2

Thirdly the YPAG were invited to co-design the mini-capsules’ packaging. They were given mock up capsules and a range of food boxes and packaging, and worked in groups to design how the mini-capsules would be packaged. They were asked to consider current packaging of medical devises and if there was a way of making the packaging more child friendly. They had to consider how many capsules would be packed, whether altogether, in blister packs separately or any other form they might have seen from their own experiences. Again the YPAG worked with the designers from Renfrew Group International, who attended a YPAG meeting. We worked all together on ideas that were subsequently drawn professionally and brought back to the YPAG group for discussion and feedback. The YPAG suggested separating the capsules out into three portions; each portion containing the number of mini capsules to be swallowed over the 3 days of the study. The YPAG proposed a yogurt pot packaging style, where three pots would be joined in series and each pot contained 24 mini-capsules in each. The child then simply broke one pot off each day, unpeeled the lid and used the pot to pour the contents into their mouth for swallowing. At the time, the work necessary to validate the new proposed blister pack (or yoghurt pot style) packaging for regulatory purposes could have caused delays to the clinical trial set up and approvals. Upon further consultation with the YPAG they proposed the three portions should have the ability to be stacked on top of each other and packaged together within a box which could easily fit into a child’s pocket. Figure 3 shows examples of the YPAG ideas of mini-capsules’ packaging through to designers’ sketches and to real manufacturing prototypes along with Renfrew Group International designers’ interpretation of the YPAG pouch suggestion. Upon hearing the ideas from the YPAG, the manufacturer (JEB Technologies Ltd.) subsequently changed the packaging to pouches, as this was a method that could satisfy regulatory validation more easily and met the needs proposed by the YPAG. The evidence from the YPAG meeting suggested this was a recognised packaging solution, easily usable and identifiable with children. The direct effect of the involvement is the chosen packaging system used for the Transicap™ mini capsules feasibility study. The development of the yogurt pot packaging style is currently progressing for the future product packaging needs.

### YPAG tasks – design of participant information sheets

**Fig. 4:**
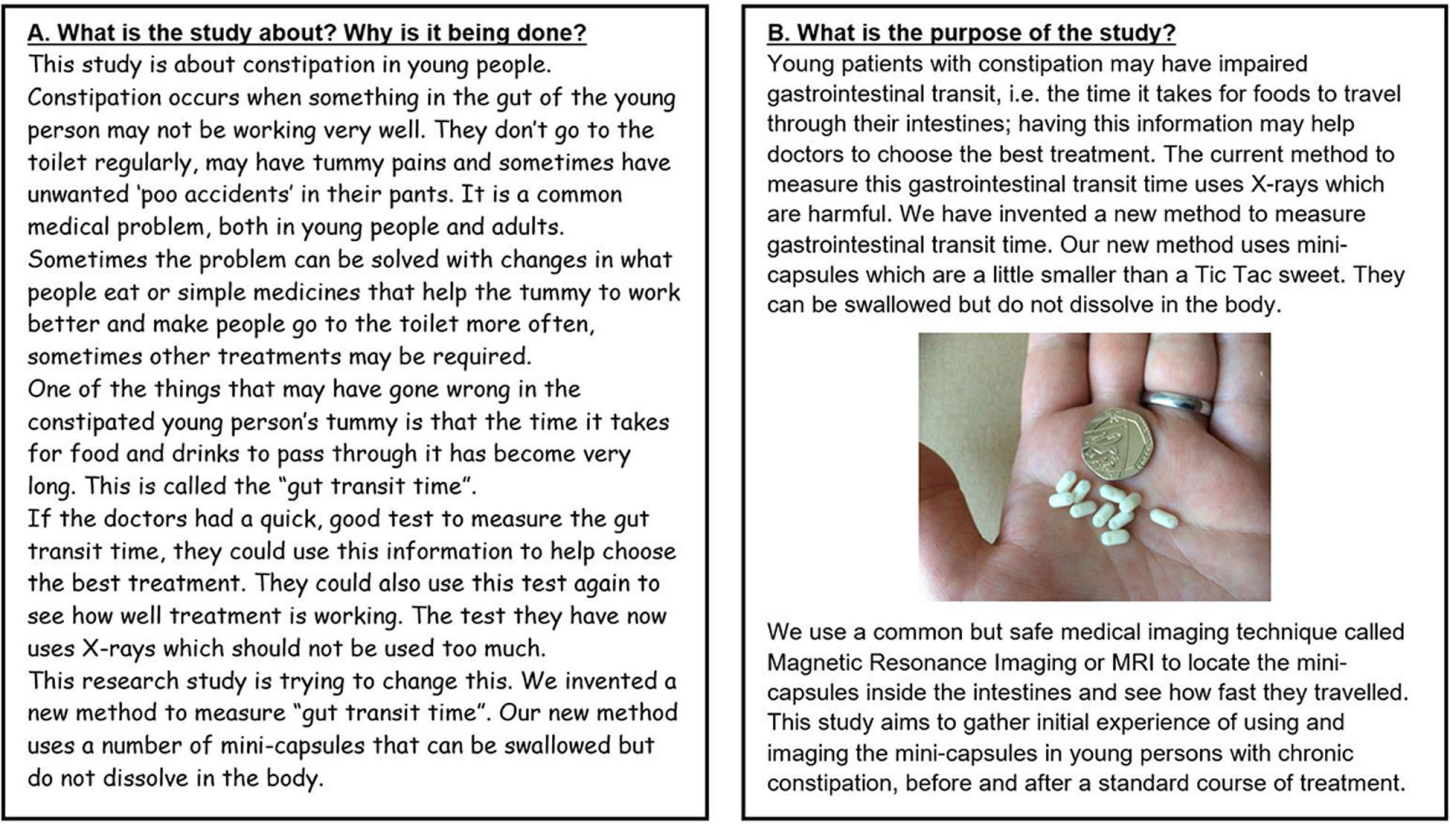
Examples of different use of language in the two patient information sheets. **a** Example from the 7–9 year olds patient information sheet. **b** Example from the 16–18 year old patient information sheet

The group were then asked to work together to devise patient and parent/carer information sheets (PIS) to be given to the MAGIC participants. They were asked to think about what information the participants and their parents/carers would need to know about the study, the language used in the information sheets and the format in which the information should be presented. A further task was to review the actual participant and parent/carer PISs developed by the researchers according to the previous discussions. They were asked to give feedback on clarity, content, layout, age groups targeted (either adult or child), use of language and appropriateness for the age groups to understand what was being asked of them, and how much information should be included in the participants PIS and their parents/carer PIS. For this purpose, some of the YPAG sessions were devoted to writing and/or reviewing patient information sheets, consent forms, assent forms, health questions etc. A suggestion by a member of the YPAG was to create separate PIS’s for different age ranges within our participant population. They felt that children should have the study explained to them at their level, rather than being presented with a generic patient information sheet written by adults for an adult audience. PIS’s were therefore written for 7–9, 10–12, 13–15 and 16–18 year olds. All documents were written by the research team then edited by the YPAG so they were worded slightly differently, increasing in complexity of language with age. The YPAG also commented on increasing the line spacing for younger patient information sheets, to make it easier for younger participants to read (Fig. 4). The PISs for the younger groups were much shorter than those for the older groups, only containing specific information which was relevant to the child. This again was a suggestion by the YPAG as they felt a younger child may have a shorter attention span and not be able to cope with reading larger documents of information. The consequences of the involvement meant our information sheets were more understandable. Both the patient information sheets and assent forms were reviewed and praised by the Ethics committee for their inclusivity.

### YPAG tasks – design of animated videos, study website and presentations

During one of our meetings the YPAG members came up with the original ideas of creating an animated video as part of the information study materials and a dedicated MAGIC study website to advertise and showcase the study. Following the suggestions on what to include in the website and how to display it, the website https://www.gastrointestinalmri.org.uk/ was created. The YPAG has their own space in the website where blogs, comments and photos of the group’s sessions are posted (having obtained written consent to display the photos upfront). For the animation video they wrote a script explaining the rationale behind the study, what was involved for the participants and instructions for the children and parents on how to take the mini capsules. The YPAG suggested the use of the animation video as it was a more modern way to explain the study to young participants. They described it as eco-friendly, accessible using phones and tablets, more memorable than reading pages of text, and may also help to overcome barriers with children with learning difficulties.

During a few sessions and with the help of student animators from Nottingham Trent University the group successfully designed, created, scripted and narrated two cartoons, now available on You Tube to explain what’s involved when volunteering for the MAGIC study. https://youtu.be/luvIutiTvr4 and https://youtu.be/w5O8lhZqEs8**.** One further suggestion by the YAPG was to include a QR code on the patient information sheets, which linked to the study’s website. The result of the YPAG involvement here meant information about the research team (and other studies the research group were involved in) could be conveyed to the studies target audience, using a modern approach which the YPAG could identify with.

The YPAG has also been involved in presenting the research. One of the Nottingham YPAG members was invited to speak at the NIHR Biomedical Research Centre conference in Nottingham in 2019 and two were invited to speak at the Nottingham Paediatric Research Showcase. The YPAG members gave a supporting talk to a large audience on the MAGIC study and their involvement and experiences of PPI. A poster co-authored by the YPAG was also presented at the British Paediatric Gastroenterologist Conference as well as a subsequent Nottingham Paediatric Research Showcase. Lastly, the YPAG contributed and are formal co-authors on the scientific paper originating from the MAGIC feasibility clinical study [1] and of course on this Commentary.

### YPAG tasks – follow-on grant application

Finally, the group were introduced to the idea of the MAGIC2 study and asked to consider the new studies proposal. They were also asked to think how they would like to continue to be involved in the future study. As a result of the YPAG reviewing the MAGIC2 protocol draft, they noted that the control arm comprising half of the participants was at a disadvantage compared to the intervention arm. In the original draft, the intervention arm underwent the test and their clinical team received the transit results so that the treatment could be reviewed including this additional new information but the control arm did not, with the intention of measuring increased treatment success when informed by the new transit test. The YPAG thought that this put the control arm participants to an unfair disadvantage, and as a result of this discussion we changed the protocol to ensure all participants, from both arms of the study received their results, with the only difference that the control arm will now receive their results but after a standard delay to allow first an appropriate comparison of treatment success between the two arms of the study. The impact of the involvement improved the study making it fairer for all participants. It is important to mention that the YPAG members are now invited to attend as full members of the follow-on NIHR MAGIC2 study grant quarterly management meetings due to their valued input throughout the development process.

### Grant funding considerations

At the start of the YPAG’s involvement with the MAGIC study, the cost of PPI was quantified and 6% of the grant funding was specifically allocated to NIHR Nottingham GenerationR YPAG. These monies were assigned to cover the cost of PPI involvement and calculated using standard tariffs which included hiring rooms, catering, appreciation vouchers for members, equipment hire and travel costs. The budget was further increased and approved at the grant stage 2. The percentage of allocated money available in the grant for PPI involvement for MAGIC2 is 37% greater than that assigned for MAGIC, due to the size and complexity of the new study, but also the increased involvement activities planned with the YPAG.

### Limitations

Our findings are based entirely on the experiences of the facilitators of the Nottingham GenerationR YPAG and the MAGIC study researchers. We never expected the YPAG to be so successful and so influential on the MAGIC study. Therefore, this Commentary is in narrative format as we did not set out to capture PPI impact or collect research data. We also did not collect data on member demographics nor reasons behind members leaving the group. During the MAGIC2 study the team plan to rectify this and address some of the gaps in data that were found during the writing of this paper. A challenge that was faced early on was that the YPAG group had to be paused due to a change in staffing. The YPAG was managed centrally by the PPI Manager within Research and Innovation Department at Nottingham University Hospitals. When this staff member left, there was not the capacity to maintain the group, which led to a break of 9 months without YPAG meetings. Management of the group is now spread across a team, rather than one individual to ensure sustainability. We would recommend considering the following to avoid this challenge; how long you would like the YPAG to be involved for; consider what will happen if a member/facilitator leaves; ensure there is adequate staff resource for the full time period of planned PPI activities.

A further limitation of our account and experience is our lack of methods for collecting formal feedback from our group. After each YPAG session feedback was obtained from those that attended, and used to continuously improve the YPAG sessions moving forward. However this wealth of data could have been collected and recorded in a more efficient and in-depth manner, providing us with qualitative measurable evidence of impact. With hindsight this is a major limitation. If we were to repeat the last 5 years of involvement with the YPAG, we would put in place systems to collect considerably more data. Going forward into MAGIC2 we plan to decide on clear parameters of what data would be beneficial to collect. On the other hand, one needs also to note that the willingness of the researchers and the CYP to work together from the first encounter allowed the rapport to be built and many positive learning, change and outcomes to develop as we went along the journey, humbly and with serendipity.

### Reflections

Staley et al. observed experiential knowledge and experience goes beyond the text book, and the issues which really matter to them are quite different from that of the clinicians [27]. Brady and Graham corroborated this statement by explaining understanding CYP are experts of their own lives, is key to meaningful and effective research [12]. With this in mind, the MAGIC study embarked on a relationship with a group of children and young people back in 2014. The researchers had a genuine interest in hearing what a group of young people could contribute to a project involving children but what the researchers could not forecast was that the MAGIC partnership with the Nottingham YPAG would last for more than 5 years and that it would continue to date. Some CYP moved on and others joined, but to our knowledge this is a unique example of a sustained collaboration throughout the life-cycle of one study and the beginning of another. There is a lot to reflect on how the research was improved by the collaboration with the YPAG and what we learned from each other.

One possible reason for success is that each YPAG session was carefully planned with the study team, based on the stage the research study was at and corresponding issues which would benefit from CYP input. The YPAG themselves were asked what activities they wanted to do at the end of each session, so they could be planned in for future sessions. This was to ensure relationships were equal and the work was meaningful both in terms of the research progress and beneficial to those involved. The quote below is from a mother who advocates the YPAG sessions her daughter has been attending: *“I’m so proud of her for not shying away in a group. YPAG is doing wonders for her confidence!”*

Building rapport with the young people is the key to their enjoyment and getting the best out of them. Allowing them to try things out and to make mistakes in a safe environment where mistakes are accepted as part of learning. Play was a big part of the YPAG sessions. Vygotsky devoted much of his life’s work to the influence of play for learning [28]. His work often forms the foundation of research and theory in cognitive development. Vygotsky theorised that community or collaboration, played a pivotal role in the “process of making meaning” in a child’s mind.

Staley and Barron said the quality of the interaction is more important than the process [16]. This was exactly true of the ‘pipework gut’ activity. Play, exploration, the physical construction of a working model, was a far more fun method of interaction which was both inclusive and memorable for everyone involved. Most importantly it suited the needs of this group of CYP, helping them to make friends with other group members and facilitators and ultimately making them feel relaxed and energised to work together on this study.

Matthews et al. reported that documents intended for the public are often not written with the public [29]. PPI provides the opportunity for the public to have their say and to be viewed by a “fresh-eyed reviewer” [30], influencing health care to their bespoke needs. The combination of clinical experience and developmental scientists when tackling a problem is not enough, they must have input from people with first-hand experience of the condition [16]. The YPAG put great effort into the writing and reviewing of patient information sheets, consent forms, assent forms and health questionnaires. This was purposefully planned to ensure all documents which would be viewed and used by the public during the MAGIC study and especially for children were written in lay terms which could be understood by all, as well as those with learning impairments. We also wanted to ensure the questions asked were relevant to patients, sensitive to their specific needs which were important to them most, and not just straight from a textbook. Barker et al. term this the “critical friend” approach, allowing the PPI to ask questions which are representative of their cohort and not of an expert panel [30].

The YPAG was involved in the production of this manuscript. During the latest meeting we have also reflected on what questions we could ask to improve the participation for our future studies. During our last session with the YPAG we put the question to them, asking what advice you would give to researchers who were embarking on a study involving CYP (Appendix 3).

## Conclusions

“Co-production” is not a quick process [31]. Planning, resourcing, staffing, executing PPI meetings and building rapport with the members is time-intensive. However, the benefits and insight which the researchers gained reflected the investment made in personal time and effort by both parties. The interactions and their experiential knowledge brought a level of reality to the study which could never be replicated from answers on anonymous questionnaires.

Our case study shows an example of a mutual partnership between researchers and a group of CYP that goes beyond theory and focuses on the patients behind the data. We’ve shown that with the right combination of involvement from CYP and researchers that it can work in practice too. We achieved a mutually beneficial partnership between the YPAG and the MAGIC study researchers. Without their contribution we would have missed a protocol flaw and been poorer in our understanding of our study participant’s lives. We have shown that CYP should be involved in research as they are valued members of our society with worthy contributions and unique insights.

## Data Availability

Not applicable.

## Appendix 1

### YPAG timeline activities, feedback and impact

**Table 2:** Feedback from the Young Persons Advisory Group (YPAG)

Date	Activity	Feedback	Impact
Sep 2014	The YPAG makes its debut on the MAGIC grant proposal. The first contact with the YPAG is mediated by the Clinical Research Network: East Midlands PPI specialist and YPAG group convenor	The YPAG agrees to participate in the MAGIC programme, 5 meetings are planned throughout the proposed programme, including meeting the technology consultants, designers, and participating at a scientific conference.	MAGIC has 6% of total cost in PPI funding (£34,053 over a grant total of £603,109) in the budget
Nov 2015	Split into 3 different groups based on age, 8–11, 12–15, 16–18. • Looked at mini capsule design • Completed tablet design questionnaire • Built gut transit model using plumbers plastic piping to educate group on study concept	The smaller, smooth and rounded shape design was preferred by all age groups, stating they would not want special tablet shape and that these could be difficult to swallow. Smooth texture would be preferred. No flavour to tablets as will not be able to please everyone with a flavour. When taking the tablets the children should have a choice about what they take this with. Recommended milkshake or squash.	Comments on capsules size, shape and characteristics were fed back to the designers. The recommendation to swallow the mini-capsules with something nice such as juice or squash was added to the draft protocol and Ethics materials.
May 2016	• Discussed the Ethics application for project • Worked together on the Patient Information Leaflets • Updated the YPAG project progress and described the next steps	Group discussed information leaflets needed to be different for age ranges, and suggested that information be divided into 3 different age brackets. Advised on wording and format of the patient information leaflets. May be more interesting if there was a video to describe the study.	Feedback from the YPAG led us to produce different Ethics information sheets divided into three age appropriate bands. They also suggested that we produce a video animation as information sheet, an idea we are working towards now.
Aug 2016	• Mini capsule packaging design - Packaging arts and crafts using food boxes and containers of different sizes and shapes • Discussed concept of creating cartoon/videos • Looked again at study information sheets	Group created packaging designs, which included splitting the tablets into three separate parts that children break off - this is so they would know what tablets had to be taken each day. Looked at stacking the containers on top of each other.	Design of packaging to include three separate portions of tablets.
Sep 2016	Device designers and technology consultant working with YPAG to finalise mini capsule design • Looked at existing packaging options based on feedback from last session. • Reviewed designs produced by manufacturers based on feedback from the last session	Group preferred the 3 different containers that were joined together but could be broken off. This would help children know how many they have to take and on which day. Can also carry them around with you with packaging that is smaller.	After session with the YPAG group agreed on the chosen design with the manufacturers.
Mar 2017	Split into age groups to provide feedback on: • The value of the vouchers which will be offered to participants • Review and provide feedback on the study advertisement posters • Answer researchers queries regarding if participants should be given the option of receiving a picture of their MRI scan • Feedback on the number of questionnaires participants would have to complete • Provide a review on the Assent Form designed for the study	Voucher: • £20 maximum – If you participate you don’t go for the money. • Give a voucher that can be used at various places, e.g. shopping centres or give the participants a list of vouchers they can choose from. Posters: • More colours and pictures (the current pictures are not related) • Less writing. • Should have more important information at the top. • Add phone number as well as email address.	The YPAG suggestions on how to answer certain queries from the REC Ethics, and also the fact that we were able to say to the REC that the YPAG had participated in developing the answers to their queries, have allowed us to pass the scrutiny and obtain full Ethics approval hence the consultation has had a great impact on the research. Have now incorporated YPAG’s suggestion in the revised version of study documentation and in the letter answering the REC Ethics queries - who praised the involvement of the YPAG.
May 2017	Students from the Nottingham Trent University attended a session to discuss the MAGIC animation video, looking to co-design the animation and script Discussion of upcoming oral presentation at Nottingham Paediatric Research Showcase Discussion on the need to hold the breath for short intervals when doing the MRI exam	The young people suggested this would be a good exercise to spend 5 min doing with the young people who would be having the MRI scans before they go in.	Breath-hold practice recommended to study participants YPAG members Anmol and Heera co-present with the investigator at the Nottingham Paediatric Research Showcase and with 2nd best oral presentation prize
Jul 2017	This session included recording animation information sheets, where the YPAG members read from a script they had co-produced for the animation. Input into the design of the MAGIC study website	Some detailed suggestions on how to display the website. YPAG member Lexie wrote a blog of the session with the animation specialist to adapt and read the scripts	The YPAG provided all the voice to be added to the animated videos, The website became a reality https://www.gastrointestinalmri.org.uk/ Many suggestions for the website were taken on board and take to the meeting of the investigator with the website designer company, including the dedicated YPAG tab where Lexie’s blog and some YPAG photos now appear
Sep 2017	The review of the animation video and feedback ready for finalisation. Started to look at script for a second video.	Only small edits proposed, a couple of script phrases were re-recorded	The animation videos were finalised and posted online https://youtu.be/luvIutiTvr4 https://youtu.be/w5O8lhZqEs8
Jan 2018	Update on MAGIC Study: • Overview of where the project is now and how the YPAG have helped to date • Work on the new follow-on grant proposal MAGIC2 starts. Discussed how the YPAG could be further involved • Discuss the need to provide a YPAG paragraph of test for article on NIHR website • Discuss poster presentation at British Paediatric Gastroenterology conference	Initial comments of clinical trial including control arm need to include the MRI test too so that no young person would miss out YPAG member Anmol collects feedback from the group and writes a paragraph for the NIHR case study on MAGIC	YPAG support to new grant proposal MAGIC2. Study protocol changes with second arm including MRI test with delayed result reading The YPAG features prominently in the ‘My research inspiration’ official case study that MAGIC has been portrayed in on the funder’s website. A poster presentation at the British Paediatric Gastroenterology conference BSPGHAN is made with YPAG’s authorship
Apr 2019	Progress report from investigator New device sachet packaging shown Plans for MAGIC stand at upcoming community event Wonder	Comments on the new device packaging collated, mostly positive, suggestion to make the writing day 1 / day 2 / day 3 on the pots more prominent Looked at possible study memento / mascot we could buy Authorship of posters and co-presentation opportunity discussed and approved	Feedback on new sachet packaging typed up and shared with manufacturer, labelling of pots have changed as a result. YPAG member Olivia co-presents with the investigator at the NIHR UK Clinical Research Facilities conference.
Jul 2019	Report back from success of MAGIC stand at Wonder community festival Focus on the start of MAGIC2 project and YPAG involvement YPAG representation on project management group	YPAG member Pavan writes a blog	YPAG’s blog uploaded to the study website. MAGIC becomes a NIHR Collaboration for Innovation case study on the NIHR website. A YPAG member attends the project’s kick-off meeting as a full member of the management team
Sep 2019	Progress update Answer specific questions on ethics sheets from the Clinical Trials Unit Look at study memento / mascot for study participants	A number of suggestions for improvement of the Ethics info sheets for the new MAGIC2 clinical trial are collected, including the use of QR codes, the addition of a certificate of participation and a separate, personal bowel diary to avoid embarrassment when discussing toilet habits	The YPAG’s suggestions are shared with the clinical trial unit to modify the information sheets for Ethics approval. MAGIC2 has 4% of total cost in PPI funding (£46,755 over a grant total of £1,186,572) in the budget. Funding for MAGIC2 PPI is increased 37% compared to MAGIC.

## Appendix 2

### Transcript of YPAG semi-structured interview carried out in 2019

#### Transcript of the young persons advisory group semi-structured interview, with the age of the members answering the questions in brackets

**Interviewer**: We have been here since 2014 with the YPAG. You have been promoting and designing our research so it is nice to be able to share our experiences over the past 5 years. Firstly can we go round and each share your impressions and opinions of what it has been like to be involved in the MAGIC Project?

**YPAG Member (11 years old):** It’s exciting to see the research developing, as the advisory group are a wide age range, I’ve really enjoying being involved and contributing to the design of the information leaflets and ethics materials.

**Interviewer:** What do you think about your breadth of involvement from assisting in designing the device to disseminating, to sitting on the management boards – do you feel like you’re doing something important?

**YPAG Member (11 years old):** It feels like we’re doing something important and its fascinating to know that people very young can help and talk to scientists and people of all age ranges can partake in research and further develop these ideas.

**Interviewer:** Do these old and important researchers know what they are doing, shall they assume that they know what children want?

**YPAG Member (15 years old):** I think being part of research is making sure that patients are pleased and satisfied with how the research is going.

**YPAG Member (17 years old):** I think it’s great that we get to meet new people and see our efforts put to good use.

**Interviewer:** How can we spread the message of research?

**YPAG Member (16 years old):** By going to schools and colleges, I think not a lot of people know that groups like the YPAG exists – when we tell people we are going to QMC people are like where are you going what are you doing? And we tell them what we are doing they are really surprised as they don’t think that people our age could be involved in research and researchers who are doing such important work – people don’t know that they have that opportunity.

**Interviewer:** Has this changed your view on what you think you might do when you grow up?

**YPAG Member (11 years old):** I would like to be a microbiologist and by taking part in the MAGIC study I feel it has helped me a lot as it has shown me the steps you may have to go through when developing a medical device.

**Interviewer:** What do you think about the YPAG meetings?

**YPAG Member (15 years old):** I like the fact that we are doing quite a lot for research as young people who think like the patients and we think from the patient’s perspective rather than the professional’s world – so it’s great to think we are helping the patients.

**YPAG Member (16 years old):** Being part of the YPAG helps you to have an insight into the other side of things – as young people we go to the doctor and get medicine and we don’t see how that medicine comes to be or any of that process. So I think I understand a lot more about how things happen.

**YPAG Member (17 years old):** We share our thoughts and its good how we can share our perspectives and evaluate and discuss pros and cons – it’s a good experience.

**YPAG Member (17 years old):** It’s good to be actually involved in a study – it’s good to interact with other people and develop yourself.

**YPAG Member (15 years old):** I think there are a lot of things that are changed because of the YPAG.

**YPAG Member (12 years old):** I think our ideas are really getting used and I think people would be surprised at how many ideas from CYP are being used in research and its really thinking that you can actually help people.

**Interviewer**: What is your favourite moment of the YPAG?

**YPAG Member (16 years old):** I think when we got to design the pills choosing the shape and the size using different boxes and colours was my favourite activity.

**YPAG Member (12 years old):** There are different activities and studies every time we meet – when you get to say what you want to say- before the YPAG I didn’t have the chance to say what I wanted to say about medical health.

**YPAG Member (17 years old):** I was quite shy when I started but then you get to know everyone and you feel comfortable.

**YPAG Member (15 years old):** There is always a chance to interact with others with ice breakers that are fun but also professionals.

**YPAG Member (16 years old):** We helped create a video to help advertise the MAGIC Study that we got to voice over and we publish our findings on social media and on the MAGIC website.

**YPAG Member (12 years old):** I think it’s a good idea for young people to share their thoughts as it can leads to improvements and change their perspective on everything.

## Appendix 3

### Notes from the 11th June virtual YPAG meeting

**Table 3:** Feedback from the Young Persons Advisory Group (YPAG) on this manuscript, on the impact of the involvement, and advice for other researchers on how to involve effectively children and young people in research. This feedback was provided at the YPAG meeting following the completion of the manuscript draft and just before submission to the journal

Discussion Topic	Considerations
**Plain English Summary Feedback**	• Well written • Really nice to see our involvement written out in a paper – validation of our involvement and that it is respected and taken on board – that it is taken seriously (15 years) • I don’t think I’ve ever seen our involvement written down like this, it’s amazing to see (15 years) • Not hard to understand, no challenging words, a good overall summary of the YPAGs involvement. (16 years) • I can’t believe we’ve been working on the MAGIC Study for 5 years! It doesn’t seem like 5 years! (16 years) • It’s a very good overview (12 years)
**Abstract**	• Thought the abstract provided a good overview (12 years) • I liked the abstract and the sub-headings – made it easy to follow (15 years)
**How has involvement in the YPAG Impacted you?**	• CONFIDENCE • MEETING NEW PEOPLE • SOCIALISING • UNDERSTANDING RESEARCH • NEW SKILLS • KNOWLEDGE • FUN! • LEARNT A LOT • PROUD • PART OF A TEAM • LEARNING JOURNEY
**Advice for Researchers**	• Do not be patronizing – the relationship needs to be equal, and built on respecting each other. • Come to the YPAG with an open mind – if you come to the group without listening and taking our ideas on board there is no point coming – do not attend to just tick a box! • Need to make sure they do not underestimate the YPAG and what we can do and contribute. • Take everyone’s point of view on board and not just your own – listen to us! • Be prepared and expect a large variety of opinions and take these on board. • Come to the YPAG as early as possible for us to be involved – at concept – and keep coming back. • My top piece of advice is to keep engaged with the young people. If we are kept in the loop of what’s happening throughout the study, we’ll be thinking about it more regularly- therefore understanding the study much better and giving us more ideas for comments to help improve the study. • Make it fun and creative. I like the idea of having an agenda so we know what to expect and also keeping us updated with any changes and the progress of their research like the MAGIC study • Spend as much time in the meetings and come back as much as possible, try not to come just once

## Abbreviations

CYP: Children and Young People
DBS: Disclosure and Barring Service
GRIPP: Guidance for reporting involvement of patients and the public
MAGIC: MAGnetic Resonance Imaging in Paediatric Constipation
MRI: Magnetic Resonance Imaging
NIHR: National Institute for Health Research
NUH: Nottingham University Hospitals
PIS: Patient Information Sheet
PPI: Public and Patient Involvement
YPAG: Young Persons Advisory Group
